# Recognition for Positive Behavior as a Critical Youth Development Construct: Conceptual Bases and Implications on Youth Service Development

**DOI:** 10.1100/2012/809578

**Published:** 2012-05-01

**Authors:** Ben M. F. Law, Andrew M. H. Siu, Daniel T. L. Shek

**Affiliations:** ^1^Department of Social Work and Social Administration, The University of Hong Kong, Pokfulam Road, Hong Kong; ^2^Department of Rehabilitation Sciences, The Hong Kong Polytechnic University, Hong Kong; ^3^Department of Applied Social Sciences, The Hong Kong Polytechnic University, Hong Kong; ^4^Public Policy Research Institute, The Hong Kong Polytechnic University, Hong Kong; ^5^Department of Social Work, East China Normal University, Shanghai, China; ^6^Kiang Wu Nursing College of Macau, Macau, China; ^7^Division of Adolescent Medicine, Department of Pediatrics, Kentucky Children's Hospital, University of Kentucky College of Medicine, Lexington, KY, USA

## Abstract

Recognition for positive behavior is an appropriate response of the social environment to elicit desirable external behavior among the youth. Such positive responses, rendered from various social systems, include tangible and intangible reinforcements. The following theories are used to explain the importance of recognizing positive behavior: operational conditioning, observational learning, self-determination, and humanistic perspective. In the current work, culturally and socially desirable behaviors are discussed in detail with reference to Chinese adolescents. Positive behavior recognition is especially important to adolescent development because it promotes identity formation as well as cultivates moral reasoning and social perspective thinking from various social systems. The significance of recognizing positive behavior is illustrated through the support, tutorage, invitation, and subsidy provided by Hong Kong's social systems in recognition of adolescent volunteerism. The practical implications of positive behavior recognition on youth development programs are also discussed in this work.

## 1. Introduction

Positive behaviors include all the observable skills that increase the likelihood of success and personal satisfaction in normative academic, work, social, recreational, community, and family settings [1]. Such behaviors aim at enhancing an individual's quality of life to ensure harmonious interaction between the individual and the environment. In adolescents, the focus can be on physical health, participation in healthy social activities, academic pursuit, getting along with family members, following cultural norms, conforming to societal standards, and contemplating on transcendental values. Significant changes in sociocognitive skills, interpersonal negotiation skills, and regulatory mechanism occur during adolescence, thus fostering the development of positive behavior [2].

Recognizing positive behavior is an appropriate response of the social environment to such behavior [3]. The ultimate aim of recognition is to encourage adolescents to continue demonstrating positive behavior. This paper discusses the following theories on positive behavior recognition: operational conditioning, observational learning, self-determination, and humanistic perspective. To fully illustrate the complexity of positive behavior, this paper describes cultural values and prosocial behavior in detail. The relationship between recognition and adolescent development, notably the formation of positive identity, is also described. The importance of recognition is then illustrated by a study of youth volunteerism in Hong Kong. This paper ends with practical implications, in which positive behavior recognition can be effectively executed in youth services.

## 2. Operant Conditioning Theory and Positive Behavior Recognition

Learning involves the acquisition of abilities that are not innate and depends on the experience and feedback from the environment [4]. Behaviorism believes that consequences trigger the repetition of behavior. Hilgard and Bower [5] established the “law of effect,” that is, if an act is followed by a favorable effect, it is likely to be repeated; conversely, the opposite leads to an unfavorable effect. Therefore, the consequences of one's present behavior play an important role in determining one's future behavior. Skinner [6] expanded Thorndike's “law of effect” by examining various types of reinforcements, punishment, reward, or punishment schedules and their respective effects on behaviors. Skinner's operant conditioning theory postulates that positive consequences for a behavior increase the likelihood of its recurrence, thereby reinforcing the relationship between behavior and the various environmental stimuli present at the time the behavior occurred.

 As a stimulus presented after an act, positive behavior recognition strengthens the occurrence of similar responses in the future, making it a kind of positive reinforcement. Recognition (or reinforcement, in this case) has two different forms according to operant conditioning theory, namely, tangible and intangible. Typical tangible reinforcements consist of rewards, including foods, drinks, and small gifts. Adolescents are subject to various positive consequences that subtly shape their behavior. Meanwhile, intangible rewards are social in nature [7]. There are two types of social reinforcements, namely, stimulation and affective rewards. Stimulation rewards include the presence of significant adults and peers, attention, and responsiveness from others, whereas affective rewards imply interpersonal warmth as manifested by respect, praise, sympathy, and affection given by individuals who are significant others.

## 3. Observational Learning and Positive Behavior Recognition

When Skinner's operant conditioning focuses solely on the consequences, Bandura's social learning theory emphasizes more on the modeling of behavior and the internal mental processes [8]. Its main axiom lies in the notion that we learn because we observe. Moreover, we learn positive behavior (e.g., normative and socially acceptable behavior) from role models and by observing the consequences of other people's behaviors [8]. When a person witnesses how positive behaviors are recognized, such as praising a classmate's good academic grades as a result of his or her hard work, the adolescent as observer would imitate the positive behavior. Moreover, the observer understands that this behavior would be recognized positively as well. Thus, recognizing positive behavior has a contagious effect: when it is recognized, observers are motivated to follow a similar track. According to social learning theory [8], observers would be attentive to the behaviors' details and their compatibility with the desired positive behavior. They are then encouraged to retain the positive behavior through their own cognitive organization, symbolic coding, and rehearsal until they are competent to perform such positive behavior, which along with necessary reinforcement, can be executed once they are ready.

 Observational learning and positive behavior recognition are highly related to shaping observers' behavior. Peers and adults discern the desired positive behavior through recognition. Once the adolescents emulate such behavior, it becomes their own, and enduring behavioral patterns are shaped.

## 4. Self-Determination Theory and Positive Behavior Recognition

Self-determination theory centers on human motivation and personality concerning people's innate growth tendencies and psychological needs. Deci and Ryan [9] proposed a continuum of motivation to human behavior, in which the two ends are intrinsic and extrinsic motivation. Intrinsic motivation is the natural, inherent drive to seek challenges and new possibilities associated with human growth and the fulfillment of psychological needs. Extrinsic motivation, meanwhile, comes from external sources. Deci and Ryan [9] derived four kinds of motivation. In “external regulation,” a behavior is performed solely due to external rewards; this is the least autonomous form of motivation. “Introjected regulation” refers to a behavior performed to preserve self-esteem and self-worth. “Identified regulation” is a mix between intrinsic and extrinsic motivation, in which a behavior is motivated by some external goals considered important by an individual. Finally, “integrated regulation” is the identification of a cluster of goals that are critical to a person's self-evaluation and life plans.

 The effect of positive behavior recognition can be entirely external if the deeds are only socially desirable and performed for the sake of external reward. The act can also be introjected because the recognition, especially from verbal praises, is associated with ego-worth; that is, a person performs socially desirable behaviors because he or she wants to be liked by external sources. However, introjected behavior remains isolated from the life tasks or personal devotions of an individual. When a person recognizes the importance of positive behavior to his or her own life tasks, he or she develops identified regulated or integrated regulation behaviors. Positive behavior recognition is still required in these two actions. Finally, a person can perform a positive behavior because he or she links such behavior with intrinsic motivation and simply enjoys it. Conceptually, the individual does not require additional external recognition for this behavior.

 Self-determination theory suggests that positive behavior recognition through external rewards can either facilitate the continuation of the behavior or prevent individuals from recognizing the meaning of such behavior. The relationship between positive behavior recognition and the use of external rewards is not direct. If the reward for an action is greater than the surface value of the positive behavior, people tend to perform the deed for sake of the external reward, which may lessen the meaning of the service and inhibit the intrinsic motivation of positive behavior. Additionally, conditioned generalized reinforcements (e.g., money) can inhibit the meaning of performing positive behavior because of the intended material gain. Thus, disproportional external rewards and conditioned generalized reinforcements can be detrimental to intrinsic motivation, especially when rewards are expected, tangible, and performance-contingent. This behavior shifts self-attributed intrinsic motivation to extrinsic recognition [9]. Therefore, positive behavior recognition should not replace the importance of the positive behavior itself [10].

## 5. Humanistic Perspective and Positive Behavior Recognition

According to the humanistic perspective, positive behavior is a sign of positive human development. As a “third force” of psychology, after psychoanalysis and behaviorism, humanistic psychology focuses on issues with higher human motives, self-development, knowledge, understanding, and aesthetics [11]. Rogers, a key figure in humanistic psychology, proposed a theory closely connected to positive behavior recognition. He stated that human behavior and experiences are guided by one basic striving in life: the fundamental tendency to develop all capacities so that the person's functioning is enhanced, thereby generating positive behavior [12]. All urges, desires, wants, goals, values, and motives are subsumed under the “organismic enhancement.” A person will become all that he or she can become with his or her potential fulfilled. One interpersonal condition that leads to healthy development is unconditional positive regard, which according to Rogers, is essential to healthy development. The absence of this condition may lead people to view themselves negatively. Human service professionals believe that the best possible conditions for personal growth are provided when people are given unconditional positive regard and acceptance.

 Unconditional positive regard is demonstrated by a person who maintains an unqualified, positive attitude towards other people, enabling its receiver to have a sense of self-worth. Positive behavior recognition by means of personal warmth can be a facet of positive regard. In addition, a significant person recognizing the good deeds of a child or adolescent with warm and supportive verbal and nonverbal gestures can initiate the individual's internal organismic enhancement, transforming them into better people throughout the process. In this way, unconditional positive regard is viewed as a type of positive behavior recognition essential for human functioning.

## 6. Antecedents of Positive Behavior Recognition

In general, the antecedents of positive behavior recognition are positive behaviors. Different types of behavior conducive to enhanced quality of life can be regarded as positive behavior. This paper focuses on two types of positive behavior, namely, cultural or societal values and prosocial behavior.

 Confucianism, the dominant ideology in the Chinese culture, aims to cultivate a supreme moral person with filial piety, interpersonal harmony, a collectivist attitude, self-fulfillment, good manners, and well-rounded education. These qualities are the indicators of a positive behavior according to the Chinese culture. Filial piety is the respectful and obliging attitude towards the elders in the family, especially the parents. Children are expected to take care of and be obedient toward their parents. Interpersonal harmony and collective decisions demonstrate the importance of person-society fit. The main themes of the Four Books of Confucianism are as follows: to “rectify one's heart; undergo self cultivation; bring one's family to unison; govern the state; and peace on earth.” Self-fulfillment is the first step of rectifying the heart by restraining personal wishes that may interfere with the collective good, while acquiring good manners is akin to undergoing self-cultivation. The Chinese emphasize the importance of education because it is the means by which to develop oneself or climb the social ladder.

 Chinese socialization practices depend on dependency, conformity, modesty, self-suppression and self-contentment training, punishment preference, shame strategy, and parent-centeredness [13]. Positive behavior within this context refers to the act of being obedient to one's parents even if it means suppressing one's personal wishes at the same time. Given that education is highly advocated for the next generation, parents encourage their children to study more. Such obedience and attentiveness to studying are two major types of positive behaviors.

 On the other hand, societal norms are expectations that guide the behavior of each member of the society [14]. Prosocial norms are ethical standards and beliefs that promote positive behavior and minimize social hazards [15]. Such norms aim to promote certain positive behaviors, such as altruism, solidarity, volunteerism, and folklore practices. These types of behavior aim to build a solid social system. For instance, schools and families have implicit and explicit proper behavioral codes, and the observance of these norms is a manifestation of positive behavior.

 Cultural and societal norms also specify socially sanctioned behavior. These norms provide sufficient information for social agents as to which behavior should be recognized or suppressed. When adolescents perform a deed in accordance with the cultural and societal codes, their behavior is viewed as positive and then recognized accordingly.

 On the other hand, prosocial behavior (e.g., offering help) is regarded as a type of universal positive behavior. Helping is essential in preserving societal solidarity, cultural value transmission, and even cultural evolution [16]. Different cultures postulate different rules of prosocial behavior. Among Chinese adolescents, for example, the push factors include the general acceptance of helping the needy among various Chinese norms [17]. Chinese culture accepts helping other people as a practice of kindness (*ren*) (Confucianism) or surrendering self-desire (Taoism), a means to alleviate inborn unhappiness or accumulate life tokens, that is, karma (Buddhism), and manifestation of nonparticularistic love (*Mozi*).

 Cultural dimensions offer antagonistic forces that act against prosocial behavior. Prosocial behavior recognition is not always rendered when pull forces are present. Two reasons for this could be the emphasis on extending help, but only to members of the group, and placing importance on studying among the Chinese. Given that the Chinese social order is hierarchical, it requires the fulfillment of responsibilities in accordance with certain major relationships in society, such as that between parent and child. As an example, the Chinese think that they must first help family members, especially their parents, before they could help outsiders. Indeed, it is filial piety that dominates the Chinese society [17]. Additionally, cultural emphasis on education implies that adolescents should spend more time studying. Chinese adolescents are more afraid of academic failure than Caucasian students [18]. Positive behavior recognition is restrained by the priority of social behavior. The various roles entailed by different types of social behavior suggest that the degree of recognition is also varied for a particular context.

## 7. Positive Behavior Recognition and Adolescent Development

Recognition for positive behavior from various social systems is crucial in adolescent development. Social agents choose the behavior to be recognized, after which the desired response is executed. Ecological perspective postulates that a behavior evolves as a function of the interplay between the person and his or her environment [19]. The environment is defined as “any event or condition outside the person that either influences or is influenced by the person (p. 232)” [20]. The person and the environment actively influence each other; moreover, “progressive, mutual accommodation between an active, growing human being and the changing properties of the immediate settings in which the developing person lives, as this process is affected by relations between these settings, and by the larger contexts in which the settings are embedded (p. 21)” [19]. Ecological perspective also emphasizes the relationship between a person and his or her environment as a constantly evolving process. Behavior is the result of the continuous decision making of a person and the response of his or her significant others to the choices made. The most critical social systems for an adolescent are family, school, and peers [20]. When an adolescent performs a positive behavior, different social agents can provide responses so that the behavior is sustained. Recognition from significant adults and peers reinforces the positive behavior.

Children and adolescents construct different forms of social knowledge, including morality and other positive behaviors, through their social experiences with adults (e.g., parents, teachers, church), peers, and siblings [21]. Affective roles in parent-child relationships have long been recognized as significant and important factors contributing to moral development and acquisition of positive behavior in children and adolescents. First, affective components of parent-child interaction, such as parental warmth, involvement, and support, are related to the development of moral reasoning. Therefore, a warm, supportive bond between parent and child may enhance the likelihood of children being motivated to listen to and respond to parental messages in the future. In addition, several parental messages suggest desirable positive behavior. Second, affective reactions are inseparable aspects of children's experiences of transgressions. Social interactions regarding moral rules, rule violations, and conflicts may be highly affectively charged. Moreover, parental affective reactions, in conjunction with reasoning, may facilitate the child's understanding and encoding of moral and social rules. Those covert transactions elaborate the various facets of positive behaviors. Recognition for positive behavior in the form of parental warmth as social reinforcement is crucial for the development of further positive behavior.

Adolescents engage in more contiguous interactions with peers than with adults [22]. Studies have shown that students prefer feedback from peers than that coming from adults, because it represents “a relatively natural consequence of friendship.” In addition, peers can “effectively model, prompt, and reinforce appropriate responses.” The positive behavior of adolescents is often a result of peer pressure. Although peer relationships seem to have become the guiding force in young people's behavior, research has found evidence that parents actually have the most significant influence on character development and decision making with regard to major issues in their children's lives [22]. Hence, parental attachment and parents' affective role are positively related to an adolescent's ability to demonstrate positive behavior. Showing attention and affection is a form of behavior recognition, which is important to adolescent development.

 According to Erikson [23], it is necessary for an adolescent to establish a unique identity. Identity formation is a constant negotiation between the social context and individual development. As a “subjective sense of an invigorating sameness continuity (p. 19)” [23], identity enables an individual to establish relationships with other people and understand oneself. The three domains of identity formation include “sense of agency,” “social relatedness,” and “political moral reasoning” [24]. Domains are more or less stable across life span development [23]. Identity formation can be realized through the interaction between the person and society. When an adolescent is recognized by his or her good behaviors, he or she is encouraged to continue to perform the behavior to the extent that his or her identity is formed. Positive behavior, such as actions that preserve prosocial norms, allows an adolescent to reflect on the society's political, moral, and historical dimensions. Thus, positive behavior recognition is closely related to adolescent development.

## 8. Impact of Recognition on Chinese Adolescents

Shek [25] investigated how cultural beliefs can affect resilience towards adversity among adolescents in Hong Kong. He found that adolescents possessing more positive Chinese cultural beliefs demonstrate stronger positive behaviors and better psychological well-being as well as manifest less behavioral problems. Thus, recognition for positive behavior reinforces the recurrence of behavior as a protective factor to adolescents.

 In a previous study focused on children's behavior at a public school in New York, Liu [26] reported that all teachers remarked that Chinese children were better behaved, more obedient, and more responsible than their Caucasian counterparts. However, instead of rewarding the children for their positive behavior, parents tended to regard this as “expected” behavior from their children that did not require recognition. Specifically, they believed that positive behavior is the duty of children and it is not necessary to reward such behavior. Cultural inhibition in this setting should be critically reevaluated.

 Wan and Salili [27] also reported that reward is generally perceived to be more effective than punishment in bringing about good behavior and in improving performance among Chinese adolescents. They found public praise to be more effective among students, than private praise in sustaining the desirable behavior.

## 9. Recognition of Positive Behavior in Action: Responses of Social Systems to Youth Volunteerism in Hong Kong

Law [28] studied the influence of family, school, and peers to youth volunteerism in Hong Kong and found that recognition for volunteer service as prosocial behavior from social systems takes various forms, including support, tutorage, invitation, and financial subsidy.

 Support for adolescent volunteers coming from members in the social systems is a form of recognition. A previous study has found that the verbal support of parents served as significant factors influencing the behavior of adolescent volunteers [29]. However, in Hong Kong, not all family members held volunteer service in high regard because it adversely affected academic performance [30]. Nevertheless, school support has been found to be conducive to adolescent volunteerism [31, 32], and peers appeared to comprise an important source of support for Hong Kong adolescents [30].

Tangible rewards for the behavior of adolescent volunteers comprise another form of recognition; such rewards can be beneficial or detrimental to further service. With reference to behaviorism, a reward is a reinforcement encouraging further action. However, it can also be detrimental because it can downgrade the service if the reward is more attractive than the service itself. Such extrinsic motivation also prevents the adolescents from experiencing intrinsic satisfaction out of the service.

Another form of recognition is tutorage, which refers to significant people teaching adolescents to perform good deeds. People from the social system might teach volunteerism to adolescents. When members of that social system actively volunteer, there is a high chance that he or she would teach adolescents how to select proper volunteering opportunities, serve the needy, and perform related skills. For example, school teachers and school social workers can train participants how to volunteer for a particular occasion.

 Another form of recognition is the invitation to further perform a positive behavior, that is, invitation to become volunteers. Invitations to become volunteers coming from family members, classmates, or peers can actually motivate adolescents to volunteer [32]. In Hong Kong, peers appear to comprise an important source of referrals for volunteers of all age groups; however, the actual effect of invitation adolescent volunteerism is not known [33]. In short, if a behavior is recognized, the social agents can invite adolescents to perform the deeds further.

 Finally, adolescents also consider financial subsidy when deciding to volunteer because participating in volunteer service requires expenses, such as those spent for transportations and meals. In Hong Kong, family and school subsidy appeared to be related to adolescent volunteerism [34]. The subsidy, if given, is a type of recognition of the social system, which highlights the importance of the activities rendered.

 All factors are related to the intention to volunteer and the volunteering behavior. Among three social systems, recognition from peers is the strongest predictor of an adolescent's intention to volunteer [28]. Hartup [35] reported that the literature on peer relationship placed more emphasis on peer deviance [36–38]. Theorists have argued that peer influence is more potent in undesirable behavior than in desirable behavior in Hong Kong [39, 40]. However, peer influence on prosocial behavior has not yet been well explored. Only few findings in peer literature have revealed that adolescents tend to prefer prosocial peers to deviant peers [41]. The relationship between “participation of positive extracurricular activities” and “positive psychological and behavioral development” is mediated by prosocial peers [42]. The finding of this study is a significant documentation of the importance of peer recognition on adolescents' volunteering intention and behavior.

 Another consistent predictor of volunteering intention and behavior is extrinsic recognition, albeit in a negative manner [28]. In volunteer service, external reward normally depends on task completion rather than performance. In this case, volunteering behavior is more attributed to extrinsic motivator than intrinsic reasons [9]. In addition, the presence of recognition in the form of extrinsic reward may lessen the meaning of service and inhibit the intrinsic motivation of volunteer service. Based on these, the conceptual foundation of recognition for positive behavior is more complex than we have initially proposed.

## 10. Implication to Positive Youth Development Programs

The curriculum plan of the Project P.A.T.H.S. focuses on the promotion of 15 positive behaviors. The project was designed according to the following 15 constructs that were found to be conducive to healthy adolescent development [43]: promotion of bonding; promotion of social, emotional, cognitive, behavioral, and moral competence; cultivation of resilience; cultivation of self-determination; promotion of spirituality; development of self-efficacy; development of a clear and positive identity; promotion of beliefs in the future; provision for positive behavior recognition; provision for opportunities for prosocial involvement; promotion of prosocial norms. The overall objective of the Tier 1 Program is to promote holistic development among junior secondary school students in Hong Kong.

 There is no particular teaching unit designated for the recognition for positive behavior in the 15 constructs, and the instructor is expected to assimilate his or her instructional strategy into all teaching units. The prerequisite of the assimilation is to adopt a strength-based perspective in youth programs [43]. In this program, instructors must focus on what the student is doing, make realistic appraisals, avoid overgeneralization, look for and give credit for evidence of progress, and positively reframe behavior. Each adolescent is viewed as a unique human being focusing on the here and now. In this manner, the instructor is able to understand that positive behavior recognition is a natural behavior during the service delivery process. The research team aims to design a range of suggested teaching strategies once the instructor has thoroughly understood the foundation of the importance of recognition. The recognition should be specific, concrete, and genuine with person relevance.

 However, instructors should be mindful of the impact of recognition. Thus, instructors must lessen the use of external rewards and use verbal praises instead. Additionally, they are encouraged to immediately cultivate the intrinsic meaning of the positive behaviors, that is, to reward students because of their efforts instead of their performance. Such recognition should enhance students' self-esteem and self-competence rather than their egos.

 Apart from adopting positive behavior recognition, instructors are also encouraged to inject the meaning of positive behavior during various activities throughout the program implementation. Adolescents should internalize the positive youth development constructs to themselves. Effective pedagogical means, such as interactive exercises and in-depth reflection, can be used so that adolescents understand the constructs more thoroughly. In this manner, positive behavior recognition is used as an auxiliary tool to reinforce learning.

## 11. Conclusion

Recognition for positive behavior is a construct for positive youth development, which deserves greater attention because of its relation to several prominent theories, including operational conditioning, observational learning, self-determination, and humanistic perspective. A supportive environment is critical in recognizing positive behavior. However, during the implementation of positive youth programs, positive behavior recognition should be used cautiously so that the surface value of recognition should not be greater than the surface value of the positive behavior. Moreover, it is necessary to help adolescents internalize other positive youth developmental constructs so that the recognition does not become the focus of program delivery.
